# Glycemic Control, Animal Protein Intake, and the Risk of Diabetic Retinopathy Progression Among Patients With Type 2 Diabetes

**DOI:** 10.1002/kjm2.70200

**Published:** 2026-04-09

**Authors:** Yu‐Ju Wu, Chih‐Cheng Hsu, Chih‐Yiu Tsai, Shang‐Jyh Hwang, Kun‐Der Lin, Pi‐Chen Lin, Ya‐Fang Huang, Chiao‐I Chang, Meng‐Chuan Huang

**Affiliations:** ^1^ Department of Nutrition and Dietetics Kaohsiung Medical University Hospital Kaohsiung Taiwan; ^2^ Graduate Institute of Medicine and Department of Public Health and Environmental Medicine, College of Medicine, School of Medicine Kaohsiung Medical University Kaohsiung Taiwan; ^3^ Institute of Population Health Sciences National Health Research Institutes Miaoli County Taiwan; ^4^ Department of Health Services Administration China Medical University Taichung Taiwan; ^5^ Department of Family Medicine Min‐Sheng General Hospital Taoyuan Taiwan; ^6^ National Center for Geriatrics and Welfare Research National Health Research Institutes Yunlin Taiwan; ^7^ Division of Endocrinology and Metabolism Chang Gung Memorial Hospital Taoyuan Taiwan; ^8^ Division of Nephrology, Department of Internal Medicine, Kaohsiung Medical University Hospital Kaohsiung Medical University Kaohsiung Taiwan; ^9^ The Lin's Clinics Kaohsiung Taiwan; ^10^ Division of Endocrinology and Metabolism, Department of Internal Medicine, Kaohsiung Medical University Hospital Kaohsiung Medical University Kaohsiung Taiwan; ^11^ Department of Public Health and Environmental Medicine, College of Medicine, School of Medicine Kaohsiung Medical University Kaohsiung Taiwan

**Keywords:** animal protein, diabetes duration, diabetic retinopathy, HbA1c, ultra‐processed food

## Abstract

Effective glycemic control and food consumption play crucial roles in modulating diabetic retinopathy (DR) progression. This observational longitudinal study explored the hemoglobin A1c (HbA1c) and dietary patterns and their associations with the risk and progression of DR among 369 individuals with type 2 diabetes. Participants were grouped by DR development as DM (no DR, *n* = 280), DR‐M (stable DR, *n* = 40), and DR‐W (worsening DR, *n* = 49) over 3 years using the American Academy of Ophthalmology DR Severity Scale. Dietary intake was evaluated using a validated food frequency questionnaire and principal component analysis. High intake was defined as a component score ≥ 2/3 and low as < 2/3. Over 3 years, HbA1c ≥ 7% and a diabetes duration > 20 years linked to a higher risk of DR‐M, with stronger effects seen with high animal protein intake. High animal protein intake was independently correlated with DR‐W (OR = 2.58, 95% CI: 1.17–5.70). A one‐unit increase in HbA1c and HbA1c ≥ 7% resulted in 2.33‐fold (95% CI: 1.08–5.06) and 8.25‐fold (95% CI: 1.64–41.43) higher risks of DR‐W, respectively, in those with high, but not low intake. Poor glycemic control and diabetes duration exceeding 20 years served as key contributors to DR‐M development and persistence over 3 years, with higher risks in strata of high animal protein intake. High animal protein intake also intensified the adverse impact of poor glycemic control on DR‐W. In summary, moderating animal protein consumption, alongside optimal glycemic management, may be relevant to DR progression. Given small DR subgroup sizes, these exploratory findings require confirmation in larger, independent cohorts.

## 1 Introduction

Diabetic retinopathy (DR) is a common microvascular complication of diabetes and a leading cause of vision loss and impairment among individuals aged 27–75 years [1]. Because the prevalence of diabetes continues to increase rapidly, approximately 191 million individuals worldwide are estimated to be diagnosed with DR by 2030; of these, > 56 million will experience vision‐threatening DR [2]. Compared with patients without DR, those with DR have higher medical expenses, require more outpatient visits, and require longer hospital stays [3]. Based on it severity, DR is classified as nonproliferative DR (NPDR) or vision‐threatening proliferative DR (PDR) [1]. A previous study found that 24.4%, 15.7%, and 8.7% of patients with diabetes had DR, NPDR, and PDR, respectively [3]. According to the Taiwan Diabetes Yearbook of 2024 [4], the number of individuals with DR has increased with the increasing number of type 2 diabetes (T2D) cases. Additionally, the number of patients with DR increased from 80,338 to 100,660, whereas the number of individuals with visual impairment or blindness increased from 8026 to 14,227.

The overall prevalence of DR increases with diabetes duration, hemoglobin A1c (HbA1c) level, and blood pressure [1]. Blood glucose control is necessary for patients with diabetes because it can reduce the risk of DR progression [5]. A 1% reduction in HbA1c is reportedly associated with 40%, 25%, and 15% decreases in the risks of DR, vision loss, and blindness, respectively [6]. Moreover. a longer diabetes duration consistently increases the risk of DR progression, even when glycemia is managed appropriately [7]. Thus, disease duration is an important factor that requires monitoring when assessing the DR risk and determining surveillance strategies.

Numerous reviews have summarized the relationship between dietary intake and DR [8, 9, 10]. Adherence to a Mediterranean diet, which is characterized by high intake of fruits, vegetables, and fish, may reportedly help protect against the development of diabetic vascular complications, including DR [11]. Contrastingly, prospective evidence regarding the effects of less healthy dietary components, such as animal protein and processed meat, on the risk of DR development is limited [12, 13]. Global dietary shifts have resulted in increased consumption of ultra‐processed foods (UPFs) [14]. Although UPFs are linked to multiple adverse health outcomes, including obesity, diabetes, and cardiovascular disease [15], their direct relationship with DR is unclear, and evidence that directly connects these foods to DR is scarce. Although research has focused on individual nutrients or food groups, the influence of overall dietary patterns and glucose‐related indicators on the risk of DR, particularly that reported by longitudinal studies, is insufficiently understood.

This observational longitudinal study examined HbA1c levels and dietary patterns including processed foods (PFs) and UPFs and their associations with the risk of DR progression in patients with T2D, including assessments based on stratified analyses.

## 2 Materials and Methods

### 2.1 Study Design and Population

This prospective study enrolled participants from the Blood Pressure Control to Reduce Renal Risk for Type 2 Diabetes Mellitus (BP4DM; ClinicalTrials.gov NCT03477786) project. This cohort study, which was initiated in January 2013 by the National Health Research Institutes, recruited patients aged 30–75 years with hypertensive T2D from the diabetes shared care program of Kaohsiung Medical University Hospital and Datong Hospital in southern Taiwan for blood pressure management interventions. This study excluded pregnant women as well as participants with type 1 diabetes, gestational diabetes, HbA1c > 10%, estimated glomerular filtration rate (eGFR) < 30 mL/min/1.73 m^2^, albuminuria > 300 mg/g, nondiabetes‐associated albuminuria, kidney stones > 0.5 cm, or a history of myocardial infarction. Additionally, individuals with a history of cerebrovascular events, foot amputation, dialysis, cirrhosis, or cancer under active treatment within 3 years were excluded. Patients received either standard blood pressure management (target blood pressure: 120/75–140/90 mmHg) or intensive blood pressure management (target blood pressure: < 120/75 mmHg). Lifestyle education and nutrition education were provided by nurses and dietitians in accordance with the diabetes shared care network education standards established by Taiwan's National Health Insurance Administration. The BP4DM intervention study concluded in December 2017.

In the present study, we included diabetic patients from the BP4DM study who had completed a full 3‐year follow‐up. The data spanned from the second year (2014) to the fifth year (2017) and comprised complete records of DR diagnosis, HbA1c measurements, and baseline dietary patterns assessed using dietary food frequency questionnaires (FFQ), totaling 369 participants for the secondary data analysis. To explore DR progression during the 3‐year follow‐up period based on the diagnostic criteria from the American Academy of Ophthalmology [16], those whose diabetic retinopathy (DR) status improved during follow‐up were excluded. Patients were categorized into the following three groups: a diabetes mellitus (DM) group without DR (*n* = 280), a maintained DR (DR‐M) group with persistent DR stage (*n* = 40), and a worsening DR (DR‐W) group with worsening DR stage (*n* = 49). Ethical review and approval were obtained from the Committee on Ethical Research of both National Health Research Institutes in Taiwan and Kaohsiung Medical University Hospital. All study participants provided written informed consent before study participation.

### 2.2 Demographic and Clinical Data Collection

Trained research assistants measured height, weight, and blood pressure. Demographic information, which was gathered through questionnaires, included age, gender, education level, diabetes duration, diabetes care status, and lifestyle factors (such as smoking, alcohol consumption, exercise, and dietary habits). Venous blood samples were obtained after 8–10 h of fasting. Urine samples were collected, maintained at 2°C–8°C, and transported to the Central Laboratory (United Medical Laboratory, Taipei, Taiwan), which is certified by the American College of Pathology and the US Laboratory Accreditation Board. Cholesterol, triglycerides, low‐density lipoprotein cholesterol (LDL‐C), high‐density lipoprotein cholesterol (HDL‐C), uric acid, and serum creatinine were analyzed using an automatic analyzer (ADVIA 1800; Siemens Healthcare, New York, NY, USA). HbA1c levels were analyzed using high‐performance liquid chromatography (Variant II; Bio‐Rad, Hercules, CA, USA).

### 2.3 Plasma Malondialdehyde Measurements

Fasting blood samples were analyzed to determine malondialdehyde (MDA) concentrations using the thiobarbituric acid (TBA) reactive substances assay (Cayman Chemical Company, Ann Arbor, MI, USA), which is a standard method of monitoring lipid peroxidation that relies on the formation of MDA‐TBA adducts under acidic and high‐temperature conditions (90°C–100°C) measured spectrophotometrically at 530 nm [17].

### 2.4 Dietary Assessment

This study utilized a validated 45‐question semi‐quantitative FFQ to evaluate participants' dietary patterns, including the frequency and portion sizes of consumed food groups, as well as certain eating behaviors [18]. Participants indicated their food consumption frequency over a 6‐month period by choosing from nine predefined frequencies that ranged from “almost never” to “four to six times per day.” The intake of specific food groups (such as staple foods, bread, cereals, root vegetables, other vegetables, fresh fruits, canned juices, fish, meat, eggs, soy‐based items, milk, and dairy products) was expressed in terms of the usual portion sizes consumed daily, weekly, or monthly. The consumption frequencies of the listed food groups were converted to weekly equivalents before estimating the daily energy intake of the participants. This estimation calculated the daily servings of food groups and cooking oils reported in the FFQ and transformed them into caloric and macronutrient contributions [18].

The international system used to classify processed foods is called the NOVA Food Classification [19], which categorizes foods into the following four groups based on the degree, nature, and purpose of processing: unprocessed or minimally processed foods; processed culinary ingredients; PFs; and UPFs. In this study, according to the NOVA classification standards, items related to the total consumption frequency of PFs and UPFs per week were selected from the FFQ. The cutoff points for distinguishing between high intake and low intake were ≥ 9.8 times per week (≥ 2/3) and < 9.8 times per week (< 2/3), respectively.

### 2.5 Statistical Analysis

Data are reported as *n* (%) or mean ± standard deviation. Pearson's chi‐squared test and an analysis of variance (ANOVA) were used to examine differences in demographic and clinical features among the DM, DR‐M, and DR‐W groups. Dietary patterns were derived from FFQ responses using principal component analysis (PCA). PCA with varimax rotation was applied to 35 food items, producing four principal components (PCs) that explained most of the overall variance. PCs with factor loading values greater than 0.3 were interpreted as distinct dietary patterns. For each retained PC, individual PC scores were calculated for each patient by summing the weighted contributions of the corresponding food items. Within each dietary pattern, high intake was defined as a principal component score ≥ 2/3 of the distribution and low intake as < 2/3. Multiple logistic regression models were conducted to determine associations between clinical and dietary variables and the risk of DR‐M or DR‐W after adjusting for confounders including age, sex, years of education (≤ 6 or > 6 years), diabetes duration (≤ 10, 11–20, or > 20 years), cohort study group (target blood pressure control: 120/75 or 140/90 mmHg), diabetes blood pressure control (< 140/90, ≥ 140 and/or ≥ 90 mmHg), triglycerides, eGFR (< 60 or ≥ 60 mL/min/1.73 m^2^), smoking status (yes or no), alcohol consumption (yes or no), physical activity (yes or no), and total energy intake. All analyses were performed using SPSS (version 22.0; SPSS, Armonk, NY, USA), and statistical significance was defined as *p* < 0.05.

## 3 Results

Of the 369 patients with T2D in this study, 280, 40, and 49 patients comprised the DM, DR‐M, and DR‐W groups (Table 1). This study found significant differences among these three groups in diabetes duration, initial HbA1c level, and systolic blood pressure (all *p* < 0.05). Regarding the PFs intake frequency classified by the NOVA system, 28.9%, 40%, and 49% of individuals in the DM, DR‐M, and DR‐W groups, respectively, consumed PFs ≥ 9.8 times per week, and these proportions were significantly different (*p* = 0.013).

**TABLE 1:** Baseline characteristics of patients with type 2 diabetes stratified by diabetic retinopathy (DR) outcomes during the 3‐year follow‐up period (*n* = 369)†.

	Overall (*N* = 369)	DR outcome during the 3‐year follow‐up			*p* ‡
		DM (*N* = 280)	DR‐M (*N* = 40)	DR‐W (*N* = 49)	
Demographic data					
Age (years)	66.5 ± 7.9	66.8 ± 8.2^a^	66.9 ± 7.6^a^	64.9 ± 6.3^a^	0.304
< 65	132 (35.8)	93 (33.2)^a^	14 (35.0)^a,b^	25 (51.0)^b^	0.056
≥ 65	237 (64.2)	187 (66.8)^a^	26 (65.0)^a,b^	24 (49.0)^b^	
Diabetes duration (years)	12.5 ± 8.3	11.6 ± 7.6^a^	18.0 ± 9.9^b^	12.8 ± 8.7^a^	< 0.001
≤ 10	187 (50.7)	152 (54.3)^a^	10 (25.0)^b^	25 (51.0)^a^	< 0.001
11–20	130 (35.2)	98 (35.0)^a^	15 (37.5)^a^	17 (34.7)^a^	
> 20	52 (14.1)	30 (10.7)^a^	15 (37.5)^b^	7 (14.3)^a^	
Gender					
Male	177 (48.0)	133 (47.5)^a^	20 (50.0)^a^	24 (49.0)^a^	0.946
Female	192 (52.0)	147 (52.5)^a^	20 (50.0)^a^	25 (51.0)^a^	
BMI (kg/m^2^)	26.6 ± 3.9	26.7 ± 3.8^a^	26.4 ± 4.3^a^	26.6 ± 3.9^a^	0.910
Education ≤ 6 years	117 (31.7)	91 (32.5)	13 (32.5)	13 (26.5)	0.705
Current smoker (%)	53 (14.4)	38 (13.6)^a^	5 (12.5)^a^	10 (20.4)^a^	0.425
Alcohol drinker (%)	34 (9.2)	26 (9.3)^a^	5 (12.5)^a^	3 (6.1)^a^	0.555
Exercise habits (%)	268 (72.6)	201 (71.8)^a^	30 (75.0)^a^	37 (75.5)^a^	0.811
Clinical data					
HbA1c (%)	7.0 ± 1.0	6.9 ± 0.9^a^	7.6 ± 1.1^b^	7.2 ± 1.1^a,b^	< 0.001
< 7%	185 (50.1)	152 (54.3)^a^	10 (25.0)^b^	23 (46.9)^a,b^	0.002
≥ 7%	184 (49.9)	128 (45.7)^a^	30 (75.0)^b^	26 (53.1)^a,b^	
HbA1c change (%) during 3 years	−0.02 ± 0.97	−0.03 ± 0.93^a^	−0.12 ± 1.07^a^	0.11 ± 1.06^a^	0.508
HbA1c stable or decrease	188 (50.9)	147 (52.5)^a^	23 (57.5)^a^	18 (36.7)^a^	0.086
HbA1c worsening	181 (49.1)	133 (47.5)^a^	17 (42.5)^a^	31 (63.3)^a^	
eGFR (mL/min/1.73m^2^)	88.5 ± 27.9	87.5 ± 27.3^a^	90.1 ± 29.7^a^	93.1 ± 30.3^a^	0.400
< 60	52 (14.1)	35 (12.5)^a^	7 (17.5)^a^	10 (20.4)^a^	0.275
≥ 60	317 (85.9)	245 (87.5)^a^	33 (82.5)^a^	39 (79.6)^a^	
Systolic BP (mmHg)	139.4 ± 17.5	138.0 ± 1.04^a^	145.2 ± 17.5^b^	142.9 ± 17.1^a,b^	0.016
Diastolic BP (mmHg)	78.1 ± 11.5	77.5 ± 11.4^a^	78.7 ± 10.0^a^	81.6 ± 12.7^a^	0.064
DM BP control (mmHg)					
< 140/90	181 (49.1)	147 (52.5)^a^	13 (32.5)^a^	21 (42.9)^a^	0.039
≥ 140/90	188 (50.9)	133 (47.5)^a^	27 (67.5)^a^	28 (57.1)^a^	
Creatinine (mg/dL)	0.88 ± 0.34	0.89 ± 0.35^a^	0.86 ± 0.26^a^	0.85 ± 0.30^a^	0.755
Uric acid (mg/dL)	5.6 ± 1.4	5.6 ± 1.4^a^	5.6 ± 1.2^a^	5.7 ± 1.3^a^	0.726
Triglycerides (mg/dL)	132.6 ± 77.0	135.9 ± 73.6^a^	121.3 ± 83.7^a^	122.7 ± 89.4^a^	0.340
Cholesterol (mg/dL)	167.6 ± 29.4	169.3 ± 29.2^a^	161.4 ± 30.9^a^	163.0 ± 28.3^a^	0.138
HDL (mg/dL)	48.1 ± 20.2	48.9 ± 22.3^a^	46.5 ± 11.6^a^	44.8 ± 10.3^a^	0.371
LDL (mg/dL)	94.9 ± 24.4	95.6 ± 24.6^a^	89.9 ± 22.0^a^	94.6 ± 25.4^a^	0.381
Malondialdehyde (μmol/L)	14.9 ± 5.0	14.6 ± 4.7^a^	15.2 ± 4.7^a^	16.1 ± 6.3^a^	0.128
Dietary data					
Energy (kcal/day)	1507.3 ± 817.9	1503.7 ± 818.0^a^	1623.9 ± 917.2^a^	1432.7 ± 734.5^a^	0.543
Animal protein pattern score		−0.03 ± 0.88^a^	0.02 ± 1.16^a^	0.16 ± 1.43^a^	0.484
Eating‐out pattern score		−0.02 ± 1.00^a^	−0.05 ± 0.88^a^	0.17 ± 1.10^a^	0.428
Salty food pattern score		−0.01 ± 0.96^a^	−0.01 ± 1.40^a^	0.06 ± 0.86^a^	0.914
Vegetable pattern score		−0.004 ± 0.98^a^	0.13 ± 0.98^a^	−0.08 ± 1.13^a^	0.616
PFs and UPFs§	8.7 ± 7.5	8.4 ± 7.4^a^	9.7 ± 7.8^a^	9.9 ± 8.0^a^	0.295
< 9.8 times	248 (67.2)	199 (71.1)^a^	24 (60.0)^a,b^	25 (51.0)^b^	0.013
≥ 9.8 times	121 (32.8)	81 (28.9)^a^	16 (40.0)^a,b^	24 (49.0)^b^	

*Note:* ^a,b,c^Different superscript letters indicate statistically significant differences (*p* < 0.05) among the three groups, as determined by the Bonferroni post hoc analysis.

Abbreviations: BMI, body mass index; DM, diabetes mellitus; DR, diabetic retinopathy; DR‐M, maintained DR; DR‐W, worsening DR; eGFR, estimated glomerular filtration rate; HbA1c, hemoglobin A1C; HDL, high‐density lipoprotein; LDL, low‐density lipoprotein, PFs, processed foods; UPFs, ultra‐processed foods.

^†^ Values are presented as the mean ± standard deviation or N (%).

^‡^ An analysis of variance (ANOVA) or chi‐square test was used to assess differences among the DM, DR‐M, and DR‐W groups.

^§^ PFs and UPFs items were classified according to the NOVA classification system [19] . These included canned meats, processed meats, processed dairy products, processed wheat/gluten products, pickled vegetables, sweetened fruit juice, sugar‐containing beverages, bread, low‐nitrogen staple foods, starchy/thickened soup and foods with dextrinized starch, cakes and cookies, sugar substitutes, sauces, fermented soy products, and fermented foods.

We identified the following four distinct dietary patterns through PCA: animal protein, eating‐out, salty food, and vegetable dietary patterns (Table 2). The animal protein dietary pattern (PC1) was characterized by frequent consumption of red or white meat, marine or freshwater fish, fatty meats, and smoked or processed meats. The eating‐out dietary pattern (PC2) included frequent consumption of restaurant meals, fried foods, starch‐thickened soups, gluten products, Chinese staple foods, and sugar‐sweetened beverages. The salty food dietary pattern (PC3) was associated with frequent intake of pickled vegetables, sauces, root vegetables, fresh fruits, and fermented products. The vegetable dietary pattern (PC4) included frequent consumption of a variety of vegetables. The scores of these four dietary patterns were not significantly different among participants in the DM, DR‐M, and DR‐W groups. Collectively, these four dietary patterns had eigenvalues greater than 1.5 and explained 25.9% of the total variance in dietary intake.

**TABLE 2:** Factor loading matrix of the four major dietary patterns identified based on the food frequency questionnaire results (frequency/week)^†^.

	Dietary pattern			
	Animal protein	Eating‐out	Salty food	Vegetable
Freshwater fish	0.686	−0.232	0.040	0.072
Marine fish	0.673	−0.208	0.115	0.012
White meat	0.653	0.257	−0.118	0.029
Red meat	0.632	0.291	−0.100	0.039
Fatty meats and skin	0.614	−0.115	0.110	−0.005
Smoked meat, salted meat	0.414	0.234	0.119	−0.044
Processed soy products	−0.271	0.162	0.206	0.116
Eating out	−0.004	0.601	−0.061	0.030
Fried food	0.021	0.588	−0.088	−0.100
Starch/thickened soup and food	0.003	0.440	0.201	0.008
Gluten products	−0.127	0.407	0.219	0.079
Seafood	0.375	0.399	0.048	−0.118
Chinese staple food	−0.075	0.348	−0.114	0.331
Sugar‐containing packaged beverages	0.034	0.303	0.032	−0.037
Canned meats	0.024	0.293	0.288	−0.147
Low nitrogen staple foods	0.087	0.272	0.197	0.006
Chinese pastries and foreign pastries	−0.027	0.225	0.033	0.219
Seeds and nut	0.167	0.212	0.189	0.105
Handshake beverages	−0.145	0.184	0.012	−0.085
Sweetened fruit juice	−0.058	0.086	0.074	−0.008
Pickled vegetables	−0.023	−0.043	0.690	0.073
Sause use	0.002	0.204	0.620	−0.061
Root food	0.054	0.004	0.438	−0.072
Fresh fruits	0.082	−0.079	0.357	−0.072
Fermented products	−0.006	0.220	0.345	0.053
Milk, yogurt	0.018	−0.071	0.262	0.119
Eggs	0.071	0.178	0.249	−0.110
Sugar substitute	−0.201	0.083	0.242	0.184
Soy products	−0.062	0.207	0.225	−0.067
Sugar‐free tea	−0.031	0.043	0.157	−0.025
Dark‐colored vegetables	0.105	−0.155	0.130	0.873
Light‐colored vegetables	0.134	−0.115	0.079	0.869
Processed dairy products	0.005	0.075	0.169	−0.255
Bread	0.092	0.002	0.171	−0.205
Low calorie snacks	0.009	−0.135	0.130	−0.145
eigenvalues	2.82	2.58	2.02	1.64
% variance explained	8.05	7.37	5.77	4.69

^†^ Food items with factor loadings greater than 0.3 for all dietary patterns were included.

The diabetes durations and baseline HbA1c levels were significant clinical determinants of retinopathy with DR‐M over the 3‐year follow‐up period among patients with T2D (Table 3). In multivariable logistic regression analyses that adjusted for potential confounders, compared with diabetes durations ≤ 10 years, diabetes durations > 20 years were strongly associated with an increased likelihood of DR‐M (adjusted odds ratio [aOR] = 7.24; 95% confidence interval [CI]: 2.58–20.31; *p* < 0.001). Patients with a diabetes duration of 11–20 years were also at increased risk for DR‐M (aOR = 2.23; 95% CI: 0.86–5.74); however, this association was not statistically significant (*p* = 0.099). Furthermore, baseline HbA1c levels ≥ 7% (vs. < 7%) were independently associated with a higher risk of DR‐M (aOR = 3.53; 95% CI: 1.47–8.47; *p* = 0.005). Among participants with DR‐W during 3 years of observation, a higher animal protein dietary score (PC1 ≥ 2/3) was significantly correlated with augmented risks (OR = 2.58; 95% CI: 1.17–5.70; *p* = 0.019). Changes in HbA1c (aOR = 1.40; 95% CI: 0.96–2.04; *p* = 0.077) and frequent consumption of PFs and UPFs (≥ 9.8 times per week) (aOR = 2.22; 95% CI: 0.96–5.13; *p* = 0.057) were marginally associated with DR‐W (Table 3).

**TABLE 3:** Associations between baseline characteristics and the risk of DR progression during the 3‐year follow‐up period according to logistic regression (*n* = 369).

	DM (*N* = 280)	DR‐M (*N* = 40)					DR‐W (*N* = 49)				
		*N*	Crude OR (95% CI)	*p*	aOR (95% CI)	*p* ^†^	*N*	Crude OR (95% CI)	*p*	aOR (95% CI)	*p* ^†^
Age	280	40	1.00 (0.96–1.04)	0.929	0.98 (0.93–1.04)	0.495	49	0.97 (0.93–1.01)	0.132	0.96 (0.91–1.01)	0.084
Gender											
Male	133	20	1		1		24	1		1	
Female	147	20	0.91 (0.47–1.76)	0.767	1.25 (0.51–3.08)	0.628	25	0.94 (0.51–1.73)	0.848	0.77 (0.36–1.67)	0.514
DM BP control (mmHg)											
< 140/90	147	13	1		1		21	1		1	
≥ 140 and/or ≥ 90	133	27	2.30 (1.14–4.63)	0.020	2.08 (0.95–4.56)	0.066	28	1.47 (0.80–2.72)	0.215	1.34 (0.68–2.62)	0.396
eGFR											
< 60	35	7	1		1		10	1		1	
≥ 60	245	33	0.67 (0.28–1.64)	0.384	0.60 (0.21–1.71)	0.336	39	0.56 (0.26–1.22)	0.142	0.50 (0.20–1.24)	0.135
Diabetes duration (years)											
≤ 10	152	10	1		1		25	1		1	
11–20	98	15	2.33 (1.01–5.39)	0.049	2.23 (0.86–5.79)	0.099	17	1.06 (0.54–2.05)	0.876	1.10 (0.51–2.34)	0.812
> 20	30	15	7.60 (3.12–18.52)	< 0.001	7.24 (2.58–20.31)	< 0.001	7	1.42 (0.56–3.58)	0.459	1.16 (0.41–3.28)	0.785
HbA1c											
< 7%	152	10	1		1		23	1		1	
≥ 7%	128	30	3.56 (1.68–7.57)	< 0.001	3.53 (1.47–8.47)	0.005	26	1.34 (0.73–2.47)	0.343	1.32 (0.62–2.79)	0.469
HbA1c change (%) during 3 years	280	40	0.90 (0.63–1.29)	0.569	1.09 (0.71–1.67)	0.694	49	1.16 (0.85–1.59)	0.343	1.40 (0.96–2.04)	0.077
PFs and UPFs (times/week)^‡^											
< 9.8 times	199	24	1		1		25	1		1	
≥ 9.8 times	81	16	1.64 (0.83–3.24)	0.157	1.44 (0.59–3.52)	0.425	24	2.36 (1.27–4.37)	0.006	2.22 (0.98–5.02)	0.057
Dietary pattern											
Animal protein											
< 2/3	192	25	1		1		29	1		1	
≥ 2/3	88	15	1.31 (0.66–2.61)	0.443	0.78 (0.31–1.95)	0.590	20	1.51 (0.81–2.81)	0.199	2.58 (1.17–5.70)	0.019
Eating‐out											
< 2/3	189	27	1		1		30	1		1	
≥ 2/3	91	13	1.00 (0.49–2.03)	> 0.99	0.70 (0.26–1.88)	0.479	19	1.32 (0.70–2.46)	0.391	1.08 (0.46–2.57)	0.857
Salty food											
< 2/3	191	27	1		1		28	1		1	
≥ 2/3	89	13	1.03 (0.51–2.10)	0.928	0.95 (0.40–2.27)	0.906	21	1.61 (0.97–2.99)	0.132	1.04 (0.48–2.25)	0.922
Vegetable											
< 2/3	188	25	1		1		33	1		1	
≥ 2/3	92	15	1.23 (0.62–2.44)	0.561	1.75 (0.75–4.10)	0.198	16	0.99 (0.52–1.89)	0.978	1.30 (0.62–2.71)	0.492

Abbreviations: aOR, adjusted odds ratio; DM, diabetes mellitus; DR, diabetic retinopathy; DR‐M, maintained DR; DR‐W, DR‐worsening; eGFR, estimated glomerular filtration rate; HbA1c, hemoglobin A1C; PFs, processed foods; SBP, systolic blood pressure; UPFs, ultra‐processed foods.

^†^ Adjusted for age, sex (male, female), education (> 6 years, ≤ 6 years), cohort study group (target blood pressure control: 120/75 mmHg or 140/90 mmHg), diabetes blood pressure control (< 140/90 mmHg, ≥ 140 mmHg and/or ≥ 90 mmHg), triglycerides, eGFR (< 60, ≥ 60), diabetes duration (≤10 years, 11–20 years, > 20 years), smoking (yes/no), drinking (yes/no), exercise (yes/no), and energy intake using multiple logistic regression to analyze associations between DM vs. DR‐M and DM vs. DR‐W.

^‡^ PFs and UPFs were classified according to the NOVA classification system. These items included canned meats, processed meats, processed dairy products, processed wheat/gluten products, pickled vegetables, sweetened fruit juice, sugar‐containing beverages, bread, low‐nitrogen staple foods, starchy/thickened soup and food with dextrinized starch, cakes and cookies, sugar substitutes, sauces, fermented soy products, and fermented foods.

We further evaluated the role of diet in modulating DR development during the 3‐year follow‐up period (Table 4). In stratified analyses, HbA1c ≥ 7% was significantly associated with a higher risk of DR‐M among participants in both the lower and higher animal protein intake groups, with stronger associations in the high intake group (aOR = 7.59; 95% CI: 1.08–53.38; *p* = 0.042) than those in the lower intake group (aOR = 3.19; 95% CI: 1.10–9.22; *p* = 0.032). Diabetes durations > 20 years were robustly associated with the risk of DR‐M, and more pronounced effects were observed particularly among participants with higher animal protein intake (PC1 ≥ 2/3) (aOR = 31.57; 95% CI: 2.25–442.17; *p* = 0.010). Regarding DR‐W, a one‐unit increase in HbA1c (changes in HbA1c) (OR = 2.33; 95% CI: 1.08–5.06; *p* = 0.032) and HbA1c ≥ 7% (OR = 8.25; 95% CI: 1.64–41.43; *p* = 0.010) were significantly associated with higher risk among participants with higher animal protein intake (PC1 ≥ 2/3), but not among those with lower animal protein intake. Additionally, among participants who frequently consumed PFs and UPFs (≥ 9.8 times per week), HbA1c ≥ 7% was marginally associated with an increased risk of DR‐W (aOR = 3.14; 95% CI: 0.89–11.01; *p* = 0.074). In the stratified fully adjusted models, the events‐per‐variable (EPV) were approximately 1.1–2.1, reflecting the limited number of outcome events within subgroups. To address this concern, we performed sensitivity analyses using simplified models that included covariates showing some degree of association at *p* ≤ 1.00 in the multivariable analysis presented in Table 3. These simplified models increased the EPV to approximately 3.8–7.3; the direction of associations in the simplified models remained consistent with those observed in the fully adjusted models (Table S1).

**TABLE 4:** Associations between HbA1c and DR progression risk stratified by animal protein and NOVA PFs and UPFs intake during a 3‐year follow‐up period (*n* = 369).

	DM (*N* = 280)	DR‐M (*N* = 40)					DR‐W (*N* = 49)				
		*N*	Crude OR (95% CI)	*p*	aOR (95% CI)	*p* ^†^	*N*	Crude OR (95% CI)	*p*	aOR (95% CI)	*p* ^†^
< 2/3 animal protein											
HbA1c < 7%	103	7	1		1		18	1		1	
HbA1c ≥ 7%	89	18	2.98 (1.19–7.45)	0.020	3.19 (1.10–9.22)	0.032	11	0.71 (0.32–1.58)	0.397	0.71 (0.26–1.91)	0.495
HbA1c change (%) during 3 years	192	25	0.94 (0.61–1.44)	0.763	1.19 (0.69–2.04)	0.540	29	1.15 (0.79–1.68)	0.465	1.21 (0.76–1.92)	0.422
DM duration ≤ 10 years	107	8	1		1		14	1		1	
DM duration 11–20 years	68	11	2.16 (0.83–5.65)	0.115	2.31 (0.79–6.76)	0.127	11	1.24 (0.53–2.88)	0.623	1.40 (0.53–3.71)	0.498
DM duration > 20 years	17	6	4.72 (1.46–15.30)	0.010	4.35 (1.15–16.39)	0.030	4	1.80 (0.53–6.11)	0.347	2.15 (0.56–8.24)	0.265
≥ 2/3 animal protein											
HbA1c < 7%	49	3	1		1		5	1		1	
HbA1c ≥ 7%	39	12	5.03 (1.33–19.06)	0.018	7.59 (1.08–53.38)	0.042	15	3.77 (1.26–11.28)	0.018	8.25 (1.64–41.43)	0.010
HbA1c change (%) during 3 years	88	15	0.85 (0.47–1.55)	0.592	0.97 (0.48–1.98)	0.937	20	1.21 (0.69–2.14)	0.506	2.33 (1.08–5.06)	0.032
DM duration ≤ 10 years	45	2	1		1		11	1		1	
DM duration 11–20 years	30	4	3.00 (0.52–17.42)	0.221	2.33 (0.22–24.34)	0.480	6	0.82 (0.27–2.45)	0.720	0.71 (0.16–3.15)	0.648
DM duration > 20 years	13	9	15.58 (2.99–81.25)	0.001	31.57 (2.25–442.17)	0.010	3	0.94 (0.23–3.90)	0.937	0.16 (0.02–1.19)	0.073
< 9.8 times PFs and UPFs^‡^											
HbA1c < 7%	109	6	1		1		15	1		1	
HbA1c ≥ 7%	90	18	3.63 (1.38–9.54)	0.009	3.94 (1.33–11.67)	0.013	10	0.81 (0.35–1.88)	0.621	0.77 (0.28–2.17)	0.627
HbA1c change (%) during 3 years	199	24	1.07 (0.70–1.63)	0.760	1.31 (0.79–2.18)	0.299	25	1.26 (0.86–1.87)	0.241	1.39 (0.89–2.18)	0.150
DM duration ≤ 10 years	104	8	1		1		14	1		1	
DM duration 11–20 years	75	8	1.39 (0.50–3.86)	0.532	1.68 (0.52–5.41)	0.383	8	0.79 (0.32–1.98)	0.619	0.76 (0.26–2.16)	0.601
DM duration > 20 years	20	8	5.20 (1.75–15.48)	0.003	4.93 (1.33–18.28)	0.017	3	1.11 (0.29–4.24)	0.874	1.04 (0.23–4.71)	0.957
≥ 9.8 times PFs and UPFs^‡^											
HbA1c < 7%	43	4	1		1		8	1		1	
HbA1c ≥ 7%	38	12	3.40 (1.01–11.42)	0.048	2.85 (0.49–16.82)	0.246	16	2.26 (0.87–5.88)	0.093	3.14 (0.89–11.01)	0.074
HbA1c change (%) during 3 years	81	16	0.69 (0.39–1.23)	0.204	0.79 (0.29–2.13)	0.637	24	1.07 (0.63–1.83)	0.794	1.49 (0.77–2.88)	0.237
DM duration ≤ 10 years	48	2	1		1		11	1		1	
DM duration 11–20 years	23	7	7.30 (1.41–37.97)	0.018	10.29 (1.37–77.54)	0.024	9	1.71 (0.62–4.70)	0.300	1.94 (0.61–6.23)	0.263
DM duration > 20 years	10	7	16.80 (3.03–93.15)	0.001	45.69 (4.51–462.68)	0.001	4	1.75 (0.46–6.61)	0.412	1.34 (0.28–6.42)	0.714

Abbreviations: aOR, adjusted odds ratio; DM, diabetes mellitus; DR, diabetic retinopathy; DR‐M, maintained DR; DR‐W, worsening DR; HbA1c, hemoglobin A1C; PFs, processed foods; UPFs, ultra‐processed foods.

^†^ Adjusted for age, sex (male, female), education (> 6 years, ≤ 6 years), cohort study group (target blood pressure control: 120/75 mmHg or 140/90 mmHg), diabetes blood pressure control (< 140/90 mmHg, ≥ 140 mmHg and/or ≥ 90 mmHg), triglycerides, HbA1c change during 3 years, eGFR (< 60, ≥ 60), diabetes duration (≤ 10, 11–20, > 20 years), smoking (yes/no), drinking (yes/no), exercise (yes/no), and energy intake using multiple logistic regression to analyze the associations between DM vs. DR‐M and DM vs. DR‐W.

^‡^ PFs and UPFs were classified according to the NOVA classification system. These items included canned meats, processed meats, processed dairy products, processed wheat/gluten products, pickled vegetables, sweetened fruit juice, sugar‐containing beverages, breads, low‐nitrogen staple foods, starchy/thickened soup and food with dextrinized starch, cakes and cookies, sugar substitutes, sauces, fermented soy products, and fermented foods.

## 4 Discussion

In this study, poor glycemic control (HbA1c ≥ 7%) and diabetes durations > 20 years were significantly and strongly correlated with DR development and persistence (DR‐M) over 3 years, with the risk further elevated in individuals with high animal protein intake. High animal protein intake independently elevated the risk of DR‐W; in stratified analyses, poor glycemic control was associated with a higher risk among participants with high animal protein intake, but not in those with low intake. Therefore, tailoring dietary habits, particularly reducing animal protein intake and achieving glycemic control, may be related to susceptibility to DR.

In a prior cross‐sectional study [20], poor glycemic control (HbA1c ≥ 8.5%) was linked to a higher risk of DR, particularly among those who consumed diets low in vegetables, high in animal protein, and high in PFs. Increased MDA, which is a lipid oxidation biomarker, further characterized the group with high animal protein intake.

The present longitudinal analysis suggests that baseline dietary patterns were associated with subsequent DR status and may modify the relationship between glycemic control and DR progression, rather than exerting a direct causal effect. In a cross‐sectional study [21], an unhealthy dietary pattern including sugar, sweets, chips, high‐fat dairy products, and fried foods was correlated with unfavorable retinal microvascular changes. In another study [22] of patients with diabetes, those with the highest saturated fat intake were at 2.4‐fold higher risk for DR compared with participants with the lowest intake. Notably, this association persisted among participants with good glycemic control despite the relatively low‐fat consumption of populations in Japan compared with that of populations in Western countries. An Australian study [23] revealed that the effects of saturated fat intake on the risk of DR were most evident in patients with well‐controlled diabetes (< 7% HbA1c), whereas poor glycemic control seemed to mask this dietary influence.

Protein is a cornerstone of dietary strategies for obesity management and improves glycemic control; however, the safe upper threshold for its long‐term intake is unclear [13]. Higher animal protein intake, including meat, processed meat, dairy, and fish, has been associated with increased risks of obesity and T2D in the general population [24, 25, 26, 27]; however, plant protein demonstrated neutral or protective associations [26, 28, 29]. Recent reviews have extended these findings to include complications associated with diabetes and linked meat and processed meat with an increased risk of cardiovascular disease [13]. However, prospective evidence of microvascular complications, such as DR, is limited, and their risk has largely been inferred from established factors, including hyperglycemia and hypertension. Their proposed mechanisms extend beyond saturated fat and heme iron to include preservatives, such as sodium and nitrates, which are especially abundant in processed meats. Additionally, advanced glycation end‐products in meat are particularly relevant to vascular complications associated with diabetes [13].

In rd10 mice, a short‐term lard‐based high‐fat diet (approximately 60% kcal) impaired retinal function, accelerated photoreceptor loss, and activated oxidative inflammatory pathways [30]. A systematic review of 66 rodent studies also showed that high‐fat diets (approximately 45%–60% kcal comprising lard or coconut oil) damaged retinal, retinal pigment epithelium, and vascular tissues via lipid‐driven oxidative stress, inflammation, and ceramide accumulation [31]. Such mechanisms may converge with hyperglycemia‐induced oxidative stress and inflammation, thereby compounding injury to retinal neural and vascular tissues. Mediterranean and antioxidant‐rich diets are linked to a lower risk of DR, likely because of their anti‐inflammatory and antioxidant effects [10, 11]. According to the National Health and Nutrition Examination Survey (NHANES), healthier diets are associated with reduced DR and ocular disease, and a higher oxidative balance score was associated with a 28% lower risk of retinopathy and reduced mortality [32]. These findings emphasize glycemic control and diet as key DR modifiers and support the consumption of antioxidant‐rich diets comprising low saturated fat.

Over the 3‐year follow‐up, baseline HbA1c ≥ 7% was strongly associated with a higher likelihood of DR‐M compared with HbA1c < 7%, thus underscoring poor glycemic control as a central determinant of sustained disease. Evidence from multiple populations consistently reinforced HbA1c as the strongest predictor of DR progression. Among Chinese patients, the progression risk increased sharply, even within guideline‐recommended levels, as the baseline HbA1c increased from 5.2% to 6.4% [33]. In Singapore, a higher mean HbA1c (8.2% vs. 7.3%) combined with elevated systolic blood pressure predicted moderate DR [34]. Meta‐analyses performed in Africa also highlighted poor glycemic control (HbA1c > 7%) as a consistent independent risk factor for DR, and that higher levels further increased the risk [35]. Complementary evidence observed in China indicated that other metabolic and hemodynamic factors, including longer diabetes duration, hypertension, and dyslipidemia, can accelerate disease progression; however, HbA1c is the dominant predictor [36].

A study in Japan revealed that a 1% increase in the mean HbA1c nearly doubled the risk of progression from pre‐PDR to PDR within 2 years, and that HbA1c ≥ 8.6% predicted markedly higher cumulative PDR rates at 2, 5, and 10 years [37], thereby illustrating the powerful effect of poor glycemic control and the cumulative harm of sustained hyperglycemia. Additionally, a recent meta‐analysis reported that elevated HbA1c and fasting glucose were strongly associated with the risk of DR, whereas diabetes durations > 10 years had only a marginal effect [38]. A Cochrane review of 59 studies confirmed that HbA1c and the diabetes duration were consistent predictors of progression to PDR and identified renal impairment, particularly microalbuminuria, as an additional risk factor [39]. Even among patients with normoalbuminuria, longer diabetes durations independently predicted DR, thus reinforcing duration as a key determinant [40]. Collectively, these findings highlight poor glycemic control as the strongest driver of DR progression, with disease duration and renal status adding to that risk, thereby underscoring the need for vigilant, time‐sensitive monitoring strategies. Implementing structured surveillance intervals tailored to the diabetes duration and systemic risk factors may be crucial for timely detection and intervention.

### 4.1 Study Limitations

This study had some limitations. Because of its observational design, causality could not be confirmed, and residual confounding by unmeasured lifestyle or genetic factors was possible. Dietary intake was measured only at baseline, and unmeasured dietary changes during the 3‐year follow‐up may have resulted in some exposure misclassification and variability in the observed associations. Moreover, single dietary assessment limit causal inference. Although the study incorporated multiple adjustment strategies, the relatively small sample size of some subgroups (e.g., patients with longer diabetes durations or advanced DR) may have limited the statistical power of stratified analyses. These findings should therefore be interpreted with caution and considered exploratory. Despite these limitations, we believe that the observed patterns may provide preliminary insights into potential effect by modification of diabetes duration and dietary protein source, which could inform future large‐scale studies. Finally, generalizability may have been constrained by population‐specific dietary habits and healthcare contexts.

## 5 Conclusion

Poor glycemic control and longer diabetes durations correlated with DR persistence, and their effects were stronger among individuals with high animal protein intake. High animal protein intake was associated with and appeared to accentuate the adverse impact of hyperglycemia, although these observations were based on smaller sample sizes. These findings highlight the importance of glycemic control and dietary modification, particularly reducing animal protein intake, which may alleviate the risk of DR progression. Further large‐scale prospective studies are warranted to verify these associations and create recommendations for use in clinical practice.

## Funding

This study was supported by grants from the National Science and Technology Council, Taiwan (grant number: 108‐2320‐B‐037‐031, 113‐2320‐B‐037‐014‐MY2) and Kaohsiung Medical University Hospital (KMUH 113‐3R71).

## Conflicts of Interest

The authors declare no conflicts of interest.

## Supporting information


**Table S1:** Associations between HbA1c and risk of DR progression with limited covariate adjustment (simplified model), stratified by animal protein and NOVA PFs and UPFs intake during a 3‐year follow‐up period (*n* = 369).

## Data Availability

The data that support the findings of this study are available on request from the corresponding author. The data are not publicly available due to privacy or ethical restrictions.
