# Supplementary material for: Glycemic Control, Animal Protein Intake, and the Risk of Diabetic Retinopathy Progression Among Patients With Type 2 Diabetes
Source: Kaohsiung J Med Sci. 2026 Apr 9:e70200. Online ahead of print. doi: 10.1002/kjm2.70200 (PMC13399677; doi:10.1002/kjm2.70200)
Supplement: Supplementary file 1 — Table S1: Associations between HbA1c and risk of DR progression with limited covariate adjustment (simplified model), stratified by animal protein and NOVA PFs and UPFs intake during a 3‐year follow‐up period (n = 369). [file KJM2-9999-e70200-s001.pdf]

Supplementary Table 1. Associations between HbA1c and risk of DR progression with limited covariate adjustment (simplified model), stratified by animal protein and NOVA PFs and UPFs intake during a 3-year follow-up period (n=369)

	DM (N=280)	DR-M (N=40)					DR-W (N=49)				
		N	Crude OR (95%CI)	p	aOR (95% CI)	p†	N	Crude OR (95%CI)	p	aOR (95%CI)	p†
**<2/3 animal protein**											
HbA1c <7%	103	7	1		1		18	1		1	
HbA1c ≥7%	89	18	2.98(1.19-7.45)	0.020	3.10(1.12-8.59)	0.029	11	0.71(0.32-1.58)	0.397	0.74(0.29-1.86)	0.522
HbA1c change (%) during 3 years	192	25	0.94(0.61-1.44)	0.763	1.20(0.73-1.97)	0.476	29	1.15(0.79-1.68)	0.465	1.09(0.69-1.73)	0.707
DM duration ≤10 years	107	8	1		1		14	1		1	
DM duration 11-20 years	68	11	2.16(0.83-5.65)	0.115	1.90(0.70-5.16)	0.208	11	1.24(0.53-2.88)	0.623	1.24(0.52-2.92)	0.630
DM duration >20 years	17	6	4.72(1.46-15.30)	0.010	3.96(1.178-13.29)	0.026	4	1.80(0.53-6.11)	0.347	1.99(0.57-6.95)	0.278
**≥2/3 animal protein**											
HbA1c <7%	49	3	1		1		5	1		1	
HbA1c ≥7%	39	12	5.03(1.33-19.06)	0.018	3.21(0.73-14.20)	0.125	15	3.77(1.26-11.28)	0.018	7.93(2.12-29.65)	0.002
HbA1c change (%) during 3 years	88	15	0.85(0.47-1.55)	0.592	0.94(0.51-1.73)	0.852	20	1.21(0.69-2.14)	0.506	1.85(0.98-3.48)	0.057
DM duration ≤10 years	45	2	1		1		11	1		1	
DM duration 11-20 years	30	4	3.00(0.52-17.42)	0.221	2.13(0.34-13.17)	0.418	6	0.82(0.27-2.45)	0.720	0.48(0.14-1.63)	0.239
DM duration >20 years	13	9	15.58(2.99-81.25)	0.001	10.76(1.91-60.49)	0.007	3	0.94(0.23-3.90)	0.937	0.37(0.07-1.87)	0.230
**<9.8 times PFs and UPFs‡**											
HbA1c <7%	109	6	1		1		15	1		1	
HbA1c ≥7%	90	18	3.63(1.38-9.54)	0.009	3.59(1.27-10.15)	0.016	10	0.81(0.35-1.88)	0.621	0.98(0.39-2.48)	0.962
HbA1c change (%) during 3 years	199	24	1.07(0.70-1.63)	0.760	1.25(0.79-1.99)	0.345	25	1.26(0.86-1.87)	0.241	1.26(0.82-1.94)	0.285
DM duration ≤10 years	104	8	1		1		14	1		1	
DM duration 11-20 years	75	8	1.39(0.50-3.86)	0.532	1.13(0.39-3.30)	0.821	8	0.79(0.32-1.98)	0.619	0.82(0.33-2.09)	0.685
DM duration >20 years	20	8	5.20(1.75-15.48)	0.003	3.48(1.09-11.08)	0.035	3	1.11(0.29-4.24)	0.874	1.12(0.29-4.37)	0.866
**≥9.8 times PFs and UPFs‡**											
HbA1c <7%	43	4	1		1		8	1		1	
HbA1c ≥7%	38	12	3.40(1.01-11.42)	0.048	1.96(0.45-8.63)	0.374	16	2.26(0.87-5.88)	0.093	3.12(0.99-9.87)	0.053
HbA1c change (%) during 3 years	81	16	0.69(0.39-1.23)	0.204	0.83(0.41-1.70)	0.611	24	1.07(0.63-1.83)	0.794	1.47(0.78-2.77)	0.238
DM duration ≤10 years	48	2	1		1		11	1		1	
DM duration 11-20 years	23	7	7.30(1.41-37.97)	0.018	6.27(1.17-33.58)	0.032	9	1.71(0.62-4.70)	0.300	1.50(0.52-4.31)	0.453
DM duration >20 years	10	7	16.80(3.03-93.15)	0.001	15.98(2.69-94.93)	0.002	4	1.75(0.46-6.61)	0.412	1.31(0.31-5.53)	0.709

aOR, adjusted odds ratio; DM, diabetes mellitus; DR, diabetic retinopathy; DR-M, maintained DR; DR-W, worsening DR; HbA1c, hemoglobin A1C; PFs, processed foods; UPFs, ultra-processed foods.

†Adjusted for diabetes blood pressure control (<140/90 mmHg, ≥140 mmHg and/or ≥90 mmHg), HbA1c(<7, ≥7%), HbA1c change during 3 years, and diabetes duration (≤10, 11–20, >20 years) using multiple logistic regression to analyze the associations between DM vs. DR-M and DM vs. DR-W.

‡PFs and UPFs were classified according to the NOVA classification system. These items included canned meats, processed meats, processed dairy products, processed wheat/gluten products, pickled vegetables, sweetened fruit juice, sugar-containing beverages, breads, low-nitrogen staple foods, starchy/thickened soup and food with dextrinized starch, cakes and cookies, sugar substitutes, sauces, fermented soy products, and fermented foods.
